# A Deep Learning-Based Automatic Segmentation and 3D Visualization Technique for Intracranial Hemorrhage Detection Using Computed Tomography Images

**DOI:** 10.3390/diagnostics13152537

**Published:** 2023-07-31

**Authors:** Muntakim Mahmud Khan, Muhammad E. H. Chowdhury, A. S. M. Shamsul Arefin, Kanchon Kanti Podder, Md. Sakib Abrar Hossain, Abdulrahman Alqahtani, M. Murugappan, Amith Khandakar, Adam Mushtak, Md. Nahiduzzaman

**Affiliations:** 1 Department of Biomedical Physics and Technology, University of Dhaka, Dhaka 1000, Bangladesh; 2 Department of Electrical Engineering, Qatar University, Doha 2713, Qatar; 3 Department of Medical Equipment Technology, College of Applied, Medical Science, Majmaah University, Majmaah City 11952, Saudi Arabia; 4 Department of Biomedical Technology, College of Applied Medical Sciences in Al-Kharj, Prince Sattam Bin Abdulaziz University, Al-Kharj 11942, Saudi Arabia; 5 Intelligent Signal Processing (ISP) Research Lab, Department of Electronics and Communication Engineering, Kuwait College of Science and Technology, Block 4, Doha 13133, Kuwait; 6 Department of Electronics and Communication Engineering, School of Engineering, Vels Institute of Sciences, Technology, and Advanced Studies, Chennai 600117, India; 7 Center of Excellence for Unmanned Aerial Systems (CoEUAS), Universiti Malaysia Perlis, Perlis 02600, Malaysia; 8 Clinical Imaging Department, Hamad Medical Corporation, Doha 3050, Qatar; 9 Department of Electrical & Computer Engineering, Rajshahi University of Engineering & Technology, Rajshahi 6204, Bangladesh

**Keywords:** computed tomography, intracranial hemorrhage, deep learning, convolution neural network, Dice similarity coefficient (DSC), intersection over union (IoU)

## Abstract

Intracranial hemorrhage (ICH) occurs when blood leaks inside the skull as a result of trauma to the skull or due to medical conditions. ICH usually requires immediate medical and surgical attention because the disease has a high mortality rate, long-term disability potential, and other potentially life-threatening complications. There are a wide range of severity levels, sizes, and morphologies of ICHs, making accurate identification challenging. Hemorrhages that are small are more likely to be missed, particularly in healthcare systems that experience high turnover when it comes to computed tomography (CT) investigations. Although many neuroimaging modalities have been developed, CT remains the standard for diagnosing trauma and hemorrhage (including non-traumatic ones). A CT scan-based diagnosis can provide time-critical, urgent ICH surgery that could save lives because CT scan-based diagnoses can be obtained rapidly. The purpose of this study is to develop a machine-learning algorithm that can detect intracranial hemorrhage based on plain CT images taken from 75 patients. CT images were preprocessed using brain windowing, skull-stripping, and image inversion techniques. Hemorrhage segmentation was performed using multiple pre-trained models on preprocessed CT images. A U-Net model with DenseNet201 pre-trained encoder outperformed other U-Net, U-Net++, and FPN (Feature Pyramid Network) models with the highest Dice similarity coefficient (DSC) and intersection over union (IoU) scores, which were previously used in many other medical applications. We presented a three-dimensional brain model highlighting hemorrhages from ground truth and predicted masks. The volume of hemorrhage was measured volumetrically to determine the size of the hematoma. This study is essential in examining ICH for diagnostic purposes in clinical practice by comparing the predicted 3D model with the ground truth.

## 1. Introduction

Object detection is currently used in a number of biomedical domains, and these domains often involve a number of imaging modalities. Deep learning applications have been used to classify COVID-19, MERS, and SARS. Nowadays, machine learning is one of the hottest trends in image processing. A primary objective of image processing is to qualify images by incorporating multiple attributes. It is useful to incorporate biomedical image recognition into image processing, especially in biomedical imaging. A deep learning algorithm performs object detection not only with the simplest model but also with outstanding results for recognizing images [1,2]. Deep learning applications have been used to classify COVID-19, MERS, and SARS [3] and object detection of COVID-19 using an operational segmentation network [4]. Also, machine learning and deep learning techniques have been applied for liver tumor segmentation [5] and the severity of lungs [6]. Consequently, image segmentation and object detection can be used to detect intracranial hemorrhage.

To diagnose ICH, several imaging modalities are commonly used, including computed tomography (CT), magnetic resonance imaging (MRI), and positron emission tomography-computed tomography (PET-CT). Computed tomography (CT) is commonly used to perform an instantaneous scan of the brain to detect and localize hemorrhages. Compared to MRI scanning, CT scanning provides more comprehensive volumetric and anatomical information about a patient. The quality of CT images, however, is quite challenging, particularly when examining parenchymal, ventricular, and meningeal brain tissue. Consequently, segmenting intracranial hemorrhage using machine learning algorithms can be an indispensable component of CT images. CT scans assess intracranial hemorrhages by observing density changes over time, representing clot formation, clot lysis, clot retraction, and tissue loss [7]. As delay increases morbidity and mortality among patients with severe traumatic injuries or hemorrhagic strokes, every second counts.

Recent literature reports the segmentation of objects using deep learning techniques [8]. Different methodologies and efficacies of ICH segmentation from CT scans were reported. An investigation of semantic segmentation techniques that can quantify blood loss and distinguish between six categories of cerebral hemorrhages has been proposed by Yuhang et al. [9]. Medical image segmentation was hampered by a lack of data, as data collection and annotation were challenging. To address this issue, the authors propose using a pre-trained, finely tuned U-Net model. On the test set, the highest accuracy was 94.1%, which was 10.5% better than the model trained from scratch. Segmenting complex datasets with a small amount of data demonstrates the advantages of the proposed segmentation technique. Using a series of CT scans of the brain, Mingjie et al. [10] developed a novel three-dimensional (3D) method for segmenting hemorrhage regions. This method coupled a super voxel approach for initial segmentation with three-dimensional graph cuts to divide data more precisely. Adapting the 2D segmentation of cerebral hemorrhage to a 3D approach, making greater use of the intra-frame information of CT scans, was the primary innovation of that work. Kwon et al. [11] proposed Siamese U-Net to segment the abnormal regions of ICH more precisely from patient CT images. To emphasize the convolutional properties of the abnormal regions using ICH, they incorporated the differences between the bleeding regions and the healthy template into the long skip-connection of the U-Net design. Significant enhancements were observed in the Hausdorff distance (6.81%), Dice similarity coefficient (DSC) score (9.07%), and volume percentage error (40.32%) when comparing the proposed model to the conventional U-Net model. Another method for detecting and segmenting hemorrhagic lesions was presented in [2] using deep learning algorithms. They have proposed a U-Net-based deep learning backbone to detect and segment hemorrhage strokes in CT images automatically. In the pre-processing phase, CT slices with symmetrical constraints of brain images were introduced into their model. They achieved a detection accuracy of 98.59 percent, a DSC score of 80.33 percent, and an intersection over union (IoU) of 69.19 percent. Anupama et al. [12] proposed the combination of GrabCut-based segmentation and synergistic deep learning (SDL) as a GC-SDL model and achieved better ICH identification performance. The authors used Gabor filtration to remove noise from the image, thereby improving its quality. The SDL model was then utilized in the feature extraction procedure, and finally the SoftMax layer classified various types of ICHs. The GC-SDL model obtained a sensitivity, specificity, precision, and accuracy of 94.0%, 97.78%, 95.79%, and 95.73%, respectively. Vamsi et al. [13] proposed a lightweight convolution model using VGG-16 architecture and a Random Forest algorithm and attained a DSC and accuracy of 72.92% and 97.88%, respectively. Wang et al. [14] proposed a semi-supervised model for segmenting the ICH using an inverse-sigmoid-based learning strategy, which utilized 80% of data for training and obtained a DSC score of 0.67. White Matter Fuzzy c-Means (WMFCM) was used by Gautam et al. [15] to remove components, such as the cranium. According to their methodology, they were able to achieve an average DSC score of 0.82 despite the fact that the segmentation was not based on deep learning. Apart from CT image segmentation, several research works have been conducted on brain tumor segmentation using MRI imaging, employing different state-of-the-art models. For instance, M. Balwant [16] presented a review article that explored multiple CNN networks for brain tumor segmentation. Rehman et al. [17] introduced the BrainSeg-Net, which focuses on segmenting three sub-regions: Enhancing Core (EC), Whole Tumor (WT), and Tumor Core (TC) from MRI images. The proposed BrainSeg-Net architecture demonstrates promising improvements compared to existing baseline and state-of-the-art techniques. A similar approach was adopted using the RAAGR2-Net [18]. Additionally, Wu et al. [19] proposed the De-ResUnet model for brain tissue detection. The DE-ResUnet incorporates dual encoders for both T1-weighted images and texture features to uncover hidden supplementary information. Furthermore, they developed a strengthening module that enhances the initial segmentation by specifically emphasizing brain tissue regions, guided by prior knowledge and guidance. Zhao et al. [20] employed a 4D atlas-based segmentation method using the Wilcoxon signed-rank test. The objective of their study was to develop an automatic fetal brain segmentation method using deep learning, which offers improved accuracy and reliability compared to atlas-based methods.

The objective of this experiment is to detect ICH using deep learning techniques. Hemorrhage segmentation algorithms based on supervised, semi-supervised, and image-processing have already been proposed in the literature. Obtaining a high DSC score for ICH segmentation from brain CT images is quite challenging. A multi-stage approach has been implemented to segment the ICH from the brain CT images in 2D. It is difficult for clinicians to make proper clinical decisions with good DSC without a good visualization tool. To enhance the presentation of the results, several quantitative metrics and visual representations were used in the present work. This study makes the following contributions to the body of knowledge:

- This work highlights the CT image and annotation pre-processing steps in order to obtain better segmentation performance.
- Several state-of-the-art DNNs such as U-Net, U-Net++, and FPN (Feature Pyramid Network)-based deep learning models were investigated to identify the best model for ICH segmentation from the CT images.
- This work presented a 3D visualization tool to show the hemorrhage in the CT volume predicted using the proposed technique and compare it with a ground truth hemorrhage annotated by the experts.
- The volume of the hemorrhage was also calculated from the generated mask of images.

## 2. Methodology

**Figure 1:**
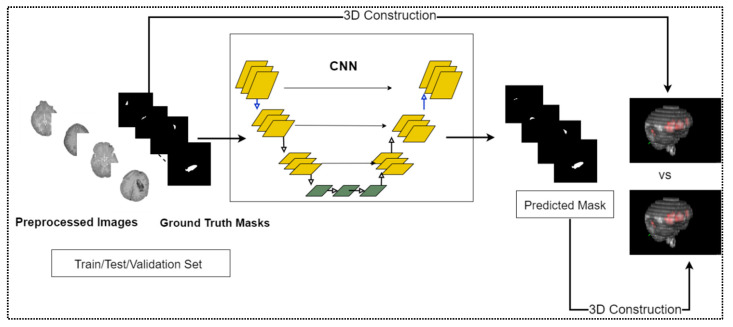
Schematic diagram of ICH detection using deep learning-driven segmentation.

A diagram illustrating the study’s overview is shown in Figure 1. As the hemorrhage is only segmented from the images, it can be considered instance segmentation. This study used a U-Net (encoder-decoder)-like convolutional neural network (CNN) to instance segment the area of hemorrhage presented in a CT image since it performed better than other deep learning networks [21].Several pre-trained models trained on the ImageNet dataset served as encoders in segmentation tasks [22].

A pre-processing step was first performed on the raw data before different variants of the U-Net model were trained to predict ICH masks. Prior to training the deep learning models, other preparatory procedures were carried out, such as dataset evaluation, data preparation, k folds cross-validation, and data augmentation.

### 2.1. Dataset Description

The dataset used in this research is a publicly available dataset published in the PhysioNet database [22]. The dataset contained 82 CT scans, in which 36 CT scans represented the five types of ICH (Epidural, Subdural, Intraventricular, Subarachnoid, and Intraparenchymal) while 46 CT volumes did not have any hemorrhage (Control). The slice number for the different CT volumes was not the same and the total number of slices from all volumes was 2814; among them, 397 were slices with ICH. The hemorrhage and skull-fracture information were recorded by two expert radiologists who delineated the intracranial hemorrhage region in each slice. The data collection maintained a multistep protocol.

**Figure 2:**
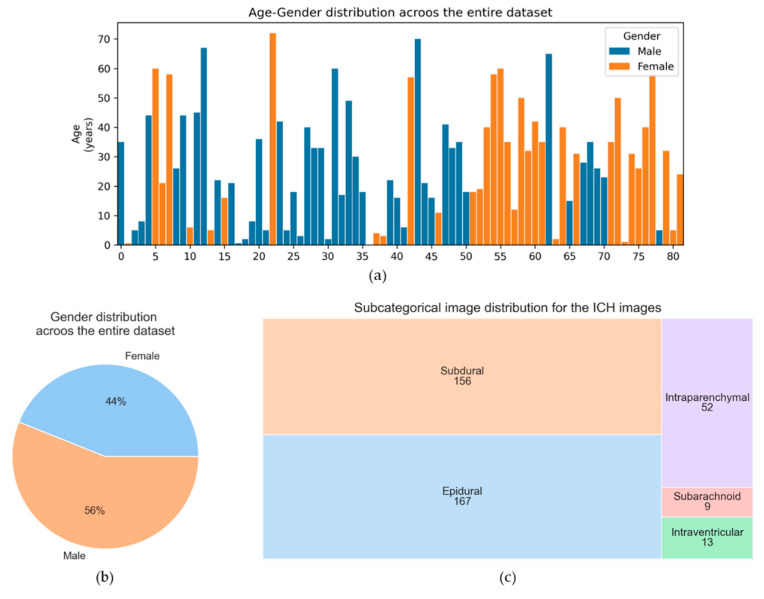
Overview of the entire dataset. (**a**) Age-gender distribution of the entire dataset, (**b**) gender distribution of the entire dataset, (**c**) sub-categorical (belongs to ICH class) image distribution for the image dataset used in this research.

The dataset provided a patient’s demographic information (.csv format) as metadata. The demographic showed the age, sex, and slice number of each patient. Most importantly, the types of hemorrhage and fracture were also listed (e.g., “1” if the hemorrhage is present; otherwise, “0”) in each slice. Figure 2 represents the overview of the entire dataset.

### 2.2. Pre-Processing Steps

**Figure 3:**
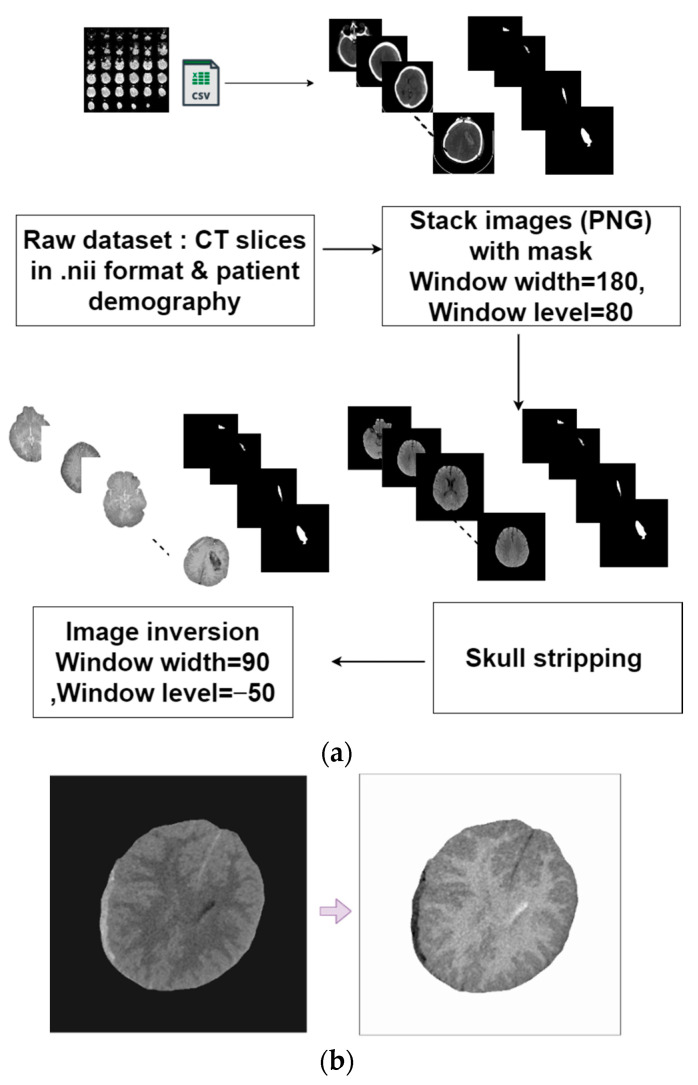
(**a**) Summary of data pre-processing, (**b**) CT image before and after inversion method.

Pre-processing data are crucial to deep learning tasks, depending on the data’s nature. It is often found that pre-processing improves the quality of images, which leads to better performance of deep learning (DL) models. Several techniques were used in this study to preprocess the dataset before training the model. Pre-processing steps for the given raw data are illustrated in Figure 3. The pre-processing techniques adopted in this study are described below:

Window level (W.L) and width (W.D) adjustment: The technique of windowing involves manipulating the grayscale component of a CT image to highlight specific structures in the anatomy. It is possible to change the brightness of an image by adjusting the window level (W.L). Contrast can be adjusted by adjusting the window width (W.D) [23]. A greater window width will show a wider range of CT values [24].

As a result, when compared with a window with a limited width, transitioning from dark to light objects takes place over a larger region. In CT number displays, the middle point of the range is known as the window level, also called the window center. CT images appear brighter when window levels are reduced, and vice versa. According to our investigations, the window width and window level were set at 180 and 80, respectively, after trial and error to make the ICH more evident in the slice. The setup was consistent across all images. As a result of these parameters, intracranial hemorrhages were clearly differentiated from other tissues in the images. Window widths and window levels, however, were readjusted to 90 and −50, respectively, during image inversion, as in this window the ICH was found most apparent in the inverted slice.

Skull-stripping: In our study, skull-stripping contributed to reducing the complexity of CT image analysis. This procedure involves removing the extracranial portion of the brain and the bone portion of the skull. However, the input images had to be converted to DICOM. To convert the raw dataset from Neuroimaging Informatics Technology Initiative (NiFTI) format to DICOM image, a Python library called NiBabel 5.1.0 was used. The idea about the skull-stripping method was adopted from Najm et al. [25]. This pre-processing was conducted using a MATLAB R2021a tool with essential functions, which are available in Github (https://github.com/WuChanada/StripSkullCT (accessed on 19 September 2022)).

Image inversion: Negative transformation is a technique used in medical image processing that plays an important role. Because it is quite challenging to detect hemorrhages from a low-contrast CT image, inverting the image may help. The image inversion is performed by “ImageJ 1.53s” software.

Figure 3 illustrates the above three steps applied to a CT image. In the brain tissue, areas containing bleeding become darker, highlighting the hemorrhage clearly. Furthermore, changing the background from black to white makes it easier to focus on the hemorrhage. The images were also adjusted for the window level and width 50 and 80, respectively, after inversion using “Imagej” software. The performance of hemorrhage recognition was significantly enhanced using these methods. To compare the performance of the framework with and without inversion, the same segmentation models were trained on both inverted and non-inverted datasets.

### 2.3. K-Fold Cross-Validation

It is important to separate training, validation, and test samples when evaluating a deep learning model so that an unbiased evaluation may be made over the entire dataset. K-fold cross-validation was the method which was utilized in this research to evaluate the DL models across the entire dataset. In this work, we used k = 5 to obtain 5-fold cross-validation. It helps the process to appraise the performance and how accurately the model is performing [26,27].

A custom-built MATLAB script was used to distribute the images and ground truth mask of different subjects among the five different folds. In each fold, 80% of the data were used as a training set and 20% of the data were used for the test set, while 20% of the training set was used for validation.

### 2.4. Data Augmentation

The number of images with hemorrhage and without hemorrhage are not equal in the dataset. There is an imbalance in the number of slices for each case. We thus have to augment the dataset to make them equally representative in the training set. It should be noted that the validation and test sets were not augmented. The validation set was employed to reduce over-fitting [6,28,29]. In this study, two specific geometric transfer functions were applied for the augmentation process: rotation with multiple angles and translation. The rotation operation can be denoted by an affine matrix:

(1) $$A_{R}=\left[\begin{array}{ccc} cos\alpha & sin\alpha & 0\\ -sin\alpha & cos\alpha & 0\\ 0 & 0 & 1 \end{array}\right]$$

Here the values of angle (α) were defined by the following:

α = {15°, 30°, 60°, 90°, 180°, 270°} (2)

The translation operation can be denoted by the following matrix:

(3) $$A_{T}=\left[\begin{array}{ccc} 1 & 0 & 0\\ 0 & 1 & 0\\ T_{x} & T_{y} & 1 \end{array}\right]$$

Here the value of $T_{x}$ and $T_{y}$ were defined by the following:

(4) $$(T_{x},\;T_{y})=\{(0.1,\;0.1),\;(0.1,-0.1),\;(-0.1,\;0.1),\;(-0.1,-0.1)\}\;$$

**Figure 4:**
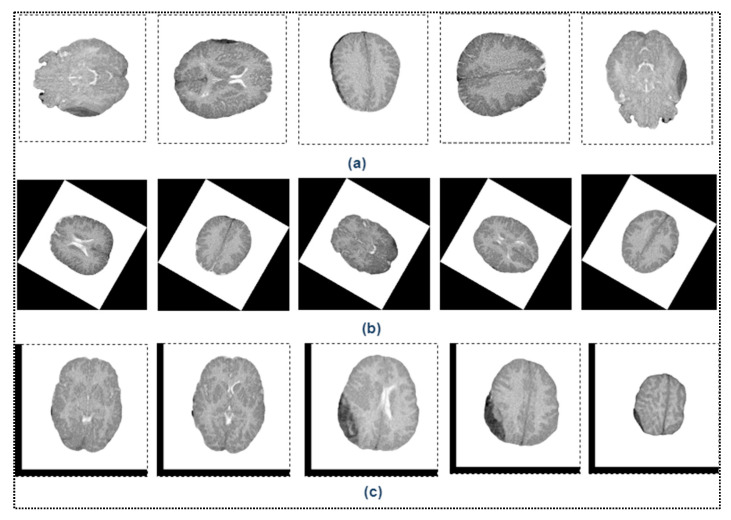
Visualization of image augmentation with different values of rotation and translation. (**a**) Rotating augmentations performed at 90°, 180°, and 270° counterclockwise. (**b**) Rotation augmentations performed at 15°, 30°, and 60° counterclockwise. (**c**) Translation.

In our training dataset, there were 9720 images generated by performing data augmentation in each training fold. Figure 4 shows samples of augmented images for different values of rotation and translation.

### 2.5. Different Image Segmentation Models

**Figure 5:**
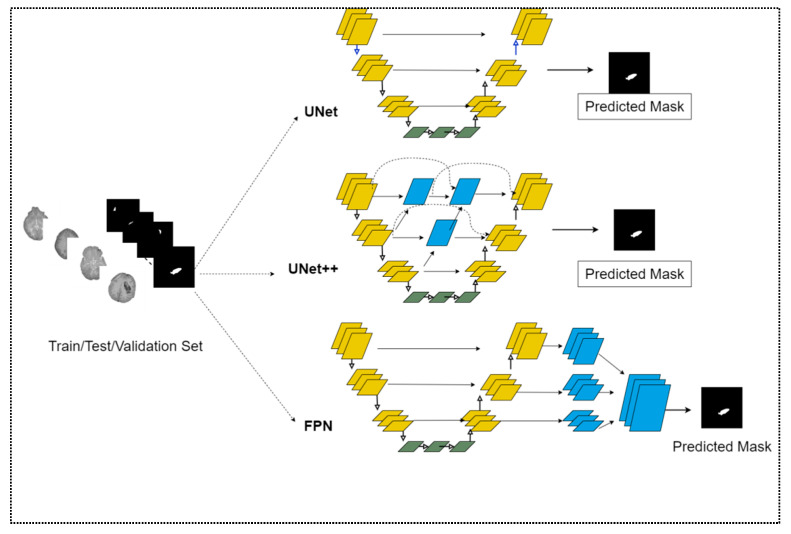
Schematic diagram of different architectures for segmentation model. The yellow and blue colored blocks represent the convolutional layer. The skip connections between the encoder and decoder are highlighted with solid and dotted arrows.

Several segmentation models with U-Net, U-Net++, and FPN architecture were used in this research. An illustration of different architectures is given in Figure 5 and briefly introduced below:

- U-Net: In deep learning, U-Net architecture is commonly used for biomedical image segmentation [30,31,32,33]. This architecture resembles the geometry of the letter “U” in its structure. Encoder or Contracting Path and Decoder or Expansion Path are its two primary elements. Encoder and decoder components both follow a symmetric path. The normalization and activation functions are used for every convolutional operation. The process commences upsampling when the transpose convolution occurs. This mechanism is in charge of producing masks.
- U-Net++: U-NET++, the updated architecture of U-Net, is frequently employed for more precise image segmentation. U-Net++ is composed of U-Nets with varying depths, but all of their decoders are linked densely and at the same resolution using newly developed skip paths. U-Net++ offers two significant enhancements over U-Net. These are:
  - Redesigning skip connections which reduce the semantic gap for ease of optimization.
  - Introducing the new technique of skip connection called Dense Skip Connection [34].
- FPN (Feature Pyramid Network): As with the previously discussed architecture, this one consists of a decoder-encoder path and follows convolutional operation. However, FPN [35] extracts the feature from the decoder at each level. This extraction contains convoluted parameters as well. The resolution of the first extracted feature is the lowest. After multiple features are generated, the resolution will progressively increase. The total dimension of features resembles a pyramid almost exactly.

#### Pre-Trained Backbone

Transfer learning is a popular method for applying the learning parameters of a model trained on a large dataset to a moderate or custom dataset. Residual Net, Inception Network, and DenseNet are prominent classification algorithms for medical image classification and were trained on the same large benchmark ImageNet dataset [36]. In our investigation, we evaluated these three segmentation architectures with 11 versions/depths of each of these models. In this work, vanila U-Net, a U-Net model with Densenet121, 161, and 201; Resnet18 and 152; and Inceptionv4 and Inceptionv2resnet pretrained encoders while U-Net++ with DenseNet201, ResNet18, and FPN with DenseNet201 pretrained encoders were evaluated.

### 2.6. Loss Function

The loss function is an essential factor for adapting the model to the collected data [37]. Forward propagation is often referred to a process of applying a dataset to a model for training. At the end of each epoch, this model produces a mask that is compared to the ground truth mask, and the error between the predicted mask and ground truth mask is calculated using the loss function and optimized using a technique called backpropagation. Consequently, backpropagation is responsible for matching the dissimilarity between a mask generated by forward propagation and the desired output. Accordingly, the model’s weights and biases are adjusted to generate new output that resembles the ground truth more closely.

The concept of loss function can be explained from an artificial neural network. If the total weight vector is *W_T_* for *x*_1_, *x*_2_, … *x_L_* number of inputs and the bias represent *θ*. The output can be expressed as:

*Y*_*L*_ = *f* (*W*_*T*_. *x* + *θ*) (5)

Now if $\hat{Y}$*_L_* represents the predicted output for the activation function of *x*, and if the targeted output denotes *Y_L_*, we can define the loss function as:

(6) $$g(W_{T},\;\theta )=\frac{1}{m}\;\sum_{i=1}^{m}L({\hat{Y}}_{L},Y_{L})$$

where *g* (average loss) was minimized by the loss function.

Several types of loss functions are used depending on the investigation, such as cross entropy loss [38], binary cross entropy, dice loss, etc. In our study, binary cross entropy (BCE) and DICE loss were used. The concept of BCE comes from the classification where the loss function is calculated at the pixel level [39,40]. The loss function, L (BCE), can be expressed as:

(7) $$L\;(\mathrm{BCE})=\;\frac{1}{m}\sum_{i=1}^{m}-(Y_{i}\;log\;(Y_{i}))+(1-Y_{i})\;(1-log(Y_{i}))$$

where *Yi* denotes the *i*th pixel of the ground truth mask and *Y*$\hat{i}$ denotes the *i*th pixel of the predicted mask. The term “*m*” denotes the maximum number of pixels in the image. On the other hand, the DICE loss is evaluated for the segmentation, and it can be expressed as a similarity index between the predicted and ground truth masks. In Equation (8), 1 is added to both the denominator and numerator to prevent the loss function from producing a zero value [40]. The dice loss L (DICE) can be expressed as:

(8) $$\;\;L\left(\mathrm{DICE}\right)\;\;=1-\frac{2\sum_{i=1}^{m}(Yi.Y\hat{i})+1}{\sum_{i=1}^{m}Yi+\sum_{i=1}^{m}Y\hat{i}+1}\;\;\;\;\;\;\;\;$$

To start, the best answer is studied for both aforementioned loss functions. However, because the BCE loss performed better than the DICE loss in the initial inquiry, the detailed analysis is conducted with BCE loss.

### 2.7. Evaluation Matrices

In this study, several parameters such as Dice similarity coefficient (DSC) (e.g., F1 score), accuracy, and intersection over union (IoU) were evaluated, which determine how well the model can predict. The accuracy, DSC, and IoU can be introduced as follows:

(9) $$\mathrm{Accuracy}=\frac{TP+TN}{TP+TN+FP+FN}$$

(10) $$\mathrm{Intersection}\;\mathrm{over}\;\mathrm{Union}\;\left(\mathrm{IoU}\right)=\frac{TP}{TP+FP+FN}$$

(11) $$\mathrm{Dice}\;\mathrm{Similarity}\;\mathrm{Coefficient}\;\left(\mathrm{DSC}\right)=\frac{2TP}{2TP+FP+FN}$$

where, *TP*, *TN*, *FP*, *FN* represent true positive, true negative, false positive, and false negative, respectively.

### 2.8. Volumetric Representation of ICH

The slices of ground truth mask for an individual head CT scan were reconstructed three dimensionally and compared in volumetric representation for predicted and ground truth masks for the sake of comparison. Three major techniques are employed to construct 3D models from 2D series of images. The data are displayed in 3D using data visualization techniques. These are: i. volume rendering (VR), ii. multi-planner rendering (MPR), and iii. surface rendering (SR) [41].

**Figure 6:**
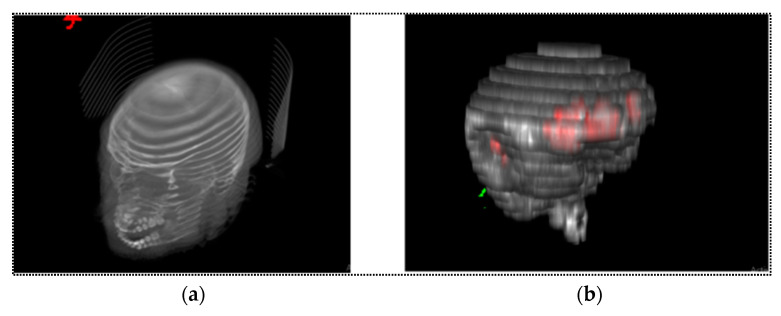
Volumetric rendering: (**a**) head slices including skull and brain and (**b**) after skull-stripping from the same patient’s CT images (The intracranial hemorrhage is highlighted with red color).

It was observed from initial experiments that volume rendering showed better visual output. Therefore, volume rendering outputs were reported in the rest of the article. The studies relied heavily on ImageJ, a powerful open-source medical image-processing software [42]. Showing the predicted mask from the segmentation model as a 3D model allows the computation of the volume of hemorrhage in volumetric perspective and will help clinicians to understand the 3D morphology of the bleed inside the brain. Figure 6 demonstrates the volume rendering technique from CT images with the head before and after skull-stripping.

**Figure 7:**
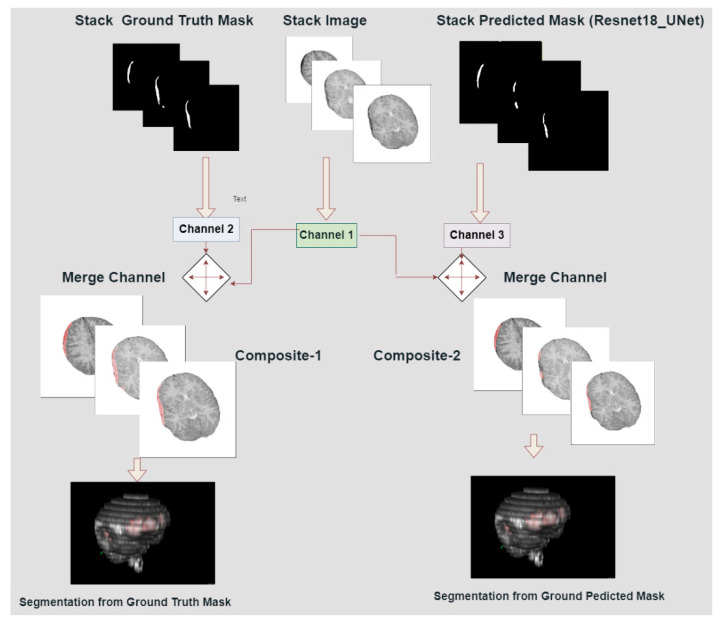
Development of 3D brain models from two different composites.

The overlaying process was carried out for the predicted & ground truth masks, overlaying it with corresponding images. The composit-1 was created by merging with channel 1 and channel 2 (highlighted in red color) while composite-2 was created by merging with channel 1 and channel 3 (highlighted in red color) which are shown in Figure 7. Then overlaid images created a stack which was intended to construct 3D models.

### 2.9. Measurement of the Volume of Hemorrhage

This study proposes the measurement of the bleed detected in CT images. The volumetric concept of the predicted mask was compared to the ground truth mask. However, the dataset contains a binary mask which indicates intracranial hemorrhage. We can consider the hemorrhage part of the masks as the region of interest (ROI). Each mask has a specific ROI, which can be combined to calculate the volume of hemorrhage. We can compare the total area of the ROI between the ground truth mask and predicted mask. Consider that the area of pixel function of a single image ground truth mask in the ROI is Δ(*x*, *y*) and the ROI of the predicted mask is Δ′(*x*, *y*), as shown below:

(12) $$A(x,y)\;=\sum_{i=1}^{n}\Delta (x_{i},y_{i})$$

(13) $$A^{\prime }(x,y)=\;\sum_{i=1}^{n}\Delta^{\prime }(x_{i},y_{i})$$

- Finally, the volume was calculated for both areas by multiplying them with the CT slice thickness (5 mm [22] for this study). Now if the slice thickness is denoted as µ, we can express the volumes as follows:

(14) $$V(x,y)=µ.\;A(x,y)=µ\;\sum_{i=1}^{n}\Delta (x_{i},y_{i})$$

(15) $$V^{\prime }(x,y)=µ.\;A^{\prime }(x,y)=µ\;\sum_{i=1}^{n}\Delta^{\prime }(x_{i},y_{i})$$

The series of masks for a particular patient where the individual ROI was marked and computed was created using a tool called a wand-tracing tool in ImageJ software [42] (https://imagej.nih.gov/ij/docs/guide/146-19.html (accessed on 21 August 2022)). It traced the sharp edge of the mask and measured the mean, max, and area of the ROI. Based on the study, we kept the unit of physical dimension for our images in ‘mm × mm’. The field of view (FOV) can be measured using a formula as:

Pixel Size = (FOV (mm))/(Matrix size) (16)

In this study, the FOV had been selected as 256 mm × 256 mm, where the matrix size (resolution) was converted to 512 × 512. Thus, the pixel size was 0.5 mm, which lies between the ranges, as mentioned in [43]. Table 1 represents the prerequisite parameters for measuring intracranial hematoma.

### 2.10. Experimental Setup

#### 2.10.1. ICH Segmentation Model

In our investigation, the training, validation, and testing of the models were conducted using a Python-based deep learning framework using the PyTorch library (version 2.0). The deep learning models were trained on Tesla T4 GPU, which is available in Google Colab. In our experiment, we used Adam as the optimizer function with a learning rate of 0.0001 and a batch size of 8, as shown in Table 2. 

We trained the model for 20 epochs and used an early stopping criterion (training will stop when there is no improvement in validation loss for 5 consecutive epochs).

#### 2.10.2. 3D Construction of Brain and ICH

There are many assistive tools that can generate 3D images from 2D series of images. ImageJ software contains several plugins that can pre-process, generate, and calculate the quantities for 3D models from 2D series of images. Table 3 summarizes the experimental setup for the 3D construction of ICH using ImageJ software. The experimental setup was taken to visualize a proper 3D model. The interpolation mode was used as trilinear in this study. This technique was used in several applications in the literature [44,45,46] for constructing a 3D model from CT scans. The sampling value was taken as 1.0 to obtain faster simulation. In addition, the investigations showed optimal 3D visualization by regulating the parameters as mentioned in Table 3.

## 3. Results and Discussion

In this study, the segmentation of ICH from the 2D CT slices was carried out using three different segmentation architectures (U-Net, U--Net++, and FPN) and eleven different segmentation models with different variants of Resnet, DenseNet, and inception models. These models were evaluated by DSC score, IoU, accuracy, and loss coefficient. In the following sub-section, the results of different models are reported, which is followed by sub-sections to introduce the volumetric representation of ICH and finally the volume measurement of intracranial hematoma.

### 3.1. Intracranial Hemorrhage Segmentation

The detection procedures were accomplished based on segmenting the mask for ICH. This approach was employed to detect the hemorrhage portion from the images using 2D segmentation pipelines for several pre-trained models, as mentioned earlier. Table 4 represents the performance matrices (loss, accuracy, DSC, and IoU) of different segmentation models. The highest DSC score, 85.76%, was achieved using the Densenet201_U-Net model for 5-fold cross validation. The IoU of 84.3% for Densenet201_U-Net was also the highest among the other models investigated in this study. The model secured 1.407% better DSC than the second-highest-performing model inceptionv4_U-Net. This suggests the superiority of the Densenet201_U-Net model in hemorrhage segmentation.

**Figure 8:**
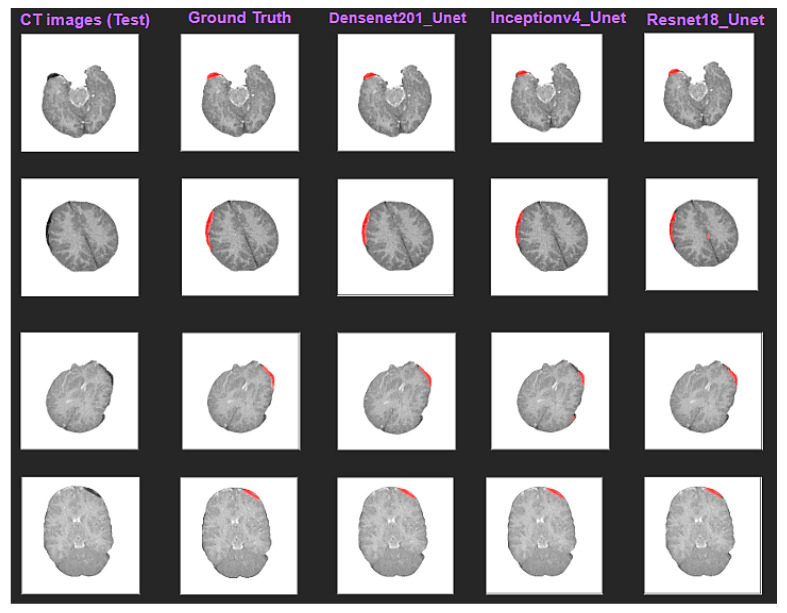
Sample CT images from test set are shown overlaying with ground truth (second row) mask of ICH and predicted mask with Densenet201_U-Net, Resnet18_U-Net, and Inceptionv4_U-Net (from third left column to fifth), respectively.

Since multiple pre-trained models were employed to investigate the performance of segmentations, the visual comparison among the predicted mask from each model was considered a qualitative evaluation process. Figure 8 shows the original CT images along with the ground truth masks and the predicted hemorrhages from three variants of U-Net architecture. The predicted region is highlighted a solid red color on the images.

### 3.2. Reconstruction of 3D Models of ICH

**Figure 9:**
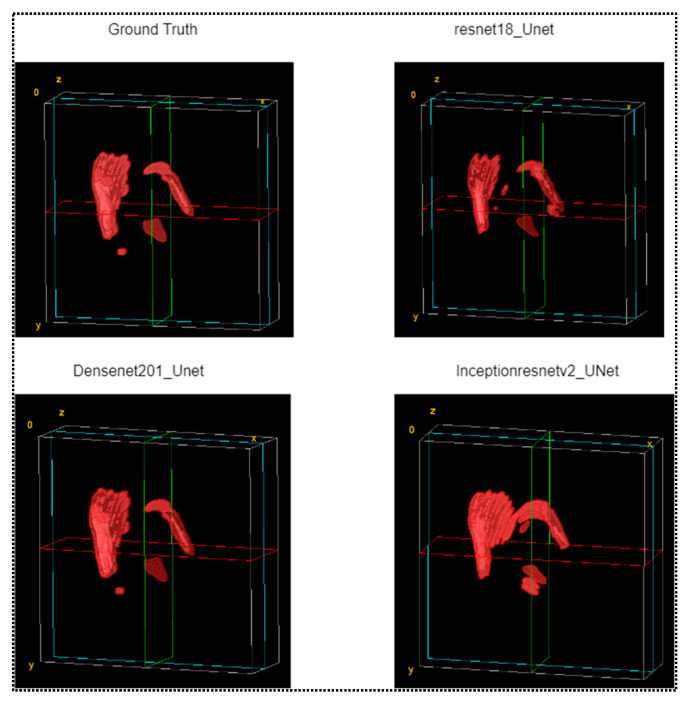
Volumetric view of ICH from ground truth mask and masked from three different predicted models.

The results of 2D segmentation generated a stack of masks from each fold of the test set, which were utilized for visualization as a volumetric model. The generated 3D models followed the same projection (Figure 9), where the xy, yz, and zx planes represent the axial, sagittal, and coronal planes, respectively, for the full brain CT images.

The entire structure of the ICH for a single patient was taken from the test fold. The predicted mask maintained the exact same serial number corresponding with the ground truth mask and CT image. Thus, it was possible to represent an entire 3D model for a single patient of intracranial hemorrhage. Figure 9 represents a volumetric view of ICH for patient no. 51.

**Figure 10:**
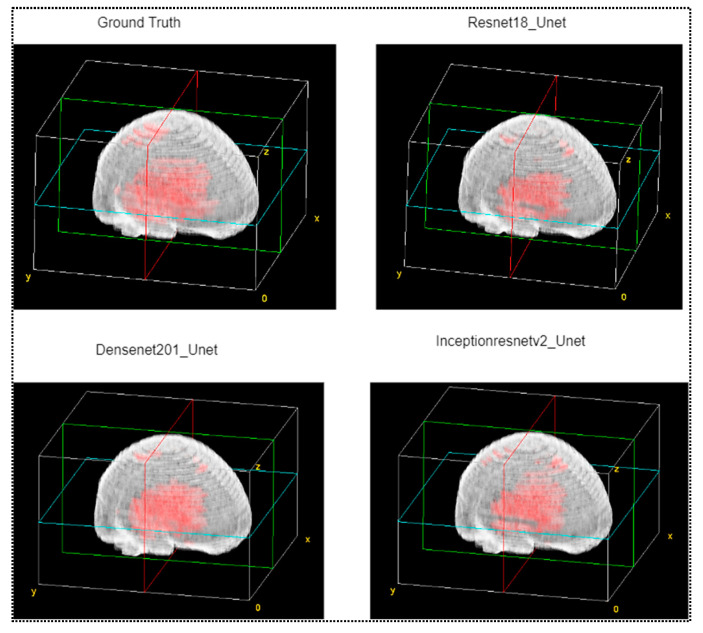
Volumetric view of full brain model overlaid with CT scans with ground truth masks and predicted masks from three best performing models.

Our investigation also examined a full brain model after merging with CT scans and corresponding masks. The simple procedure was accomplished by overlying the images with different corresponding masks (ground truth and predicted) and transfiguring it into volume viewer plugins of ImageJ. For constructing the brain models, the same parameters were applied as the ICH modeling. Figure 10 shows four different models using the volume viewer of ImageJ, where the ground truth is shown at the top left corner in the figure. A visual comparison is made for the other three best-performing models.

### 3.3. Volume Measurement of Intracranial Hematoma

This investigation measured the volume of ICH from two different masks: ground truth and predicted masks, which were predicted by the segmentation module. In this experiment, only the best-performing model, Densenet201_U-Net, was considered for the volume measurement of the intracranial hematoma. Before measuring the volume, the dimension, scales, and bit type were adjusted for all binary masks.

The volume measurement of intracranial hematoma is a sensitive task as it contains vital information of the bleed within the skull. Table 5 represents a statistical analysis between the ground truth and predicted mask in the volume measurement of the bleed in the skull for a single CT scan of an example patient (#49) from the dataset. The average area of the hematoma in the ground truth was found to be 190.15 mm^2^, while 228.9 mm^2^ was found in the predicted mask. Also, volume, median of ROI, and standard deviation were calculated for both ground truth and predicted masks. Table 5 shows that 20.04% of error was found in the predicted masks of the Densenet201_U-Net model.

The performance of our proposed investigation was compared with the existing literature. For instance, Justin et al. [14] used 254 training samples of CT images and obtained a 67% DSC score. Their dataset was collected from the Radiological Society of North America (RSNA). On the other hand, Vamsi et al. [13] included 578 brain CT images, 463 of which were stroke images, and obtained a DSC score of 72.92%. Another comparative study performed by Li et al. [2] achieved a DSC score and IoU of 80.03% and 69.19%, resepectively, using a U-Net-based deep learning network for the automatic detection and segmentation of ICH from CT images. Gautam et al. [15] found 82% of average DSC similarity using 20 brain CT volumes using White Matter Fuzzy c-Means (WMFCM) clustering and wavelet-based thresholding. Another study was conducted by Bahaduria et al. [47] using Fuzzy c-mean clustering and a region-based contour method to segment ICH. Although their approach did not use a deep learning application, they achieved an average DSC score of 87.4%. In this study, our proposed model achieved a DSC score of 85.757% along with an IoU of 84.3%. Our proposed model outperformed the previous literature [2,13,14,15] on the basis of DSC score by 23.757%, 12.655%, 5.757%, and 3.757%, respectively. Table 6 summarizes the comparative performances of the proposed method with the recent works in the litertaure.

### 3.4. Limitations of the Proposed Model and Future Works

There were a couple of limitations we discovered during our investigation. First, our proposed DenseNet201_U-Net demonstrated superior performance over other investigated networks reported in the literature. In spite of this, it was computationally intensive and had a lengthy runtime. It often took several hours to complete the training process. Additionally, the model and its intermediate variables require a large amount of memory to be stored and processed.

In addition, the study’s 3D construction method involved additional software and manual work, which made it time-consuming.

Future research will explore improvements and variations to address these limitations and further enhance the performance of these state-of-the-art segmentation models. The first priority will be to reduce the computation complexity for dense models. As part of our study, we are also planning to analyze a large dataset and propose a new algorithm for classifying the five subtypes of intracranial hemorrhage. Furthermore, we will apply a new approach to 3D modeling that uses predicted masks for automatic construction. Finally, we plan to develop a cloud-based application to segment hemorrhages from CT slices in real time.

## 4. Conclusions

In summary, deep learning algorithms pose challenges when segmenting intracranial hemorrhages of CT scans because of low resolutions and high variables in stroke location, contrast, and shape. Through our investigations into deep learning algorithms for the segmentation of intracranial hemorrhages, we have gained crucial insight. An algorithm was designed to predict hemorrhage based on an image and then generate a binary mask that was compared with the actual mask. Our training, validation, and testing datasets were created using three pre-processing techniques (windowing, skull-stripping, and inversion). The study compared 11 state-of-the-art segmentation models based on U-Net, U-Net++, and FPN architectures based on DSC and IoU scores. According to DSC and IoU scores, our proposed model, namely Dense-Net201_U-Net, achieved 85.757% and 84.3%, respectively. Using the best-performing model, we can produce accurate ICH and brain 3D models for CT imaging that will enable us to visualize bleeding inside the brain. Additionally, intracranial hematoma volumes can be measured from CT images to calculate bleed volumes, and this information can be used for longitudinal studies to track a patient’s condition over time. The results of this study demonstrate the capability of deep learning algorithms for accurately segmenting intracranial hemorrhages, providing clinicians with valuable tools for quantifying and visualizing hemorrhage volumes in CT images.

## Figures and Tables

**Table 1:** Prerequisite parameters for measuring intracranial hematoma.

Parameters	Value
Resolution	512 × 512
Physical dimension (mm × mm)	256 × 256
Method	Wand tracing
Slice thickness (mm)	5

**Table 2:** Details of ICH segmentation model training parameters.

Parameters	Value
Batch size	8
Learning rate	0.0001
Optimizer function	Adam
Number of epochs	20
Loss type	Dice loss
Number of folds	5
Learning factor	0.2
Early stopping epochs	5

**Table 3:** Experimental setup for 3D construction of ICH using ImageJ software.

Parameters	Value
Feature	Volume viewer 2.0
Method of interpolation	Trilinear
Z-aspect	3
Sampling value	1
Transfer function	Linear
Global alpha offset	50% ± 3%
Object color (ICH)	Red
Ambient, diffuse, specular, and shine	50% ± 5%

**Table 4:** Performance matrices to compare the performance of different state-of-the-art segmentation models for 2D segmentation.

Network	Loss	Acc. (%)	IoU (%)	DSC (%)
U-Net	0.73385	99.9	79.87	81.275
DenseNet201_U-Net	0.41253	99.91	84.3	85.76
DenseNet161_U-Net	0.003	99.92	81.48	82.91
DenseNet121_U-Net	0.4276	99.89	76.69	78.3
ResNet18_U-Net	0.0027	99.93	81.04	82.1
ResNet152_U-Net	0.0321	99.64	81.69	79.52
InceptionV4_U-Net	0.0027	99.93	83.03	84.35
InceptionV2-ResNet_U-Net	0.0029	99.93	78.99	80.37
DenseNet201_U-Net++	0.7036	99.77	68.01	70.14
ResNet18_U-Net++	0.614	99.88	79.9	81.47
DenseNet201_FPN	0.8018	99.93	84.09	84.09

**Table 5:** Statistical analysis of hematoma contained from ground truth mask and predicted mask.

Parameters	Ground Truth Mask (GT)	Predicted Mask (PM)	% of Error = |PM − GT|/GT × 100
Total Area (mm^2^)	1901.05	2289	20.04%
Volume (mm^3^)	9505.25	11,445	
Median of ROI (mm^2^)	161.9505	204.5	
Standard deviation	86.85599677	149.566373	
Average Area of hematoma (mm^2^)	190.105	228.9	

**Table 6:** Comparison of the proposed method with existing literatures.

Authors	Methodology and Approach	Metric (%)
Wang et al. [14,47]	Semi-supervised multitask attention-based U-Net	DSC = 67
Vamsi et al. [13]	Lightweight deep learning-based neural network	DSC = 72.92
Li et al. [2]	U-Net-based deep learning for hemorrhage detection and segmentation	DSC = 80.03 IoU = 69.19
Gautam et al. [15]	Automatic segmentation using WMFCM clustering	Average DSC = 82
Bahaduria et al. [47]	ICH detection using fuzzy c-means and region-based contour method	Average DSC = 87.4
**Proposed**	**Automatic ICH segmentation using DenseNet201_U-Net**	**DSC = 85.757** **IoU = 84.3**

## Data Availability

The dataset used in this study can be made available upon a reasonable request to the corresponding authors.
